# Characterizing the impact of podophyllotoxin on pulmonary toxicity and gut-lung microbiota interactions in SD rats based on TEC concept

**DOI:** 10.1128/spectrum.01653-24

**Published:** 2025-04-25

**Authors:** Min Wang, Xiao Ma, Junjie He, Jiaxing Sun, Feng Cai, Chuanxin Liu, Jiajia Duan

**Affiliations:** 1 Department of Respiratory and Critical Care Medicine, The First Affiliated Hospital, College of Clinical Medicine of Henan University of Science and Technology 74623, , Luoyang, China; 2 Department of Clinical Laboratory, The First Affiliated Hospital, College of Clinical Medicine of Henan University of Science and Technology 74623, , Luoyang, China; 3 School of Chinese Materia Medica, Tianjin University of Traditional Chinese Medicine 655534, Tianjin, China; 4 Department of Respiratory and Critical Care Medicine, Affiliated Hospital of Nantong University 74567, Nantong, China; 5 Henan Key Laboratory of Rare Diseases, Endocrinology and Metabolism Center, The First Affiliated Hospital, College of Clinical Medicine of Henan University of Science and Technology 74623, , Luoyang, China; Lerner Research Institute, Cleveland, Ohio, USA

**Keywords:** podophyllotoxin, pulmonary toxicity, gut microbiota, metabolome, microbiota-gut-lung axis

## Abstract

Podophyllotoxin (PPT), an extract from the traditional medicinal plant *Dysosma*, offers anti-viral and anti-cancer benefits, though its use is limited by toxicity. The mechanisms of PPT’s inherent pulmonary toxicity remain elusive. This study leverages the novel “Toxicological Evidence Chain” theory to explore the potential involvement of the “gut-lung axis” in PPT-induced pulmonary toxicity. In this study, we examined injury phenotypes in rats, evaluated pulmonary pathological changes, measured pro-inflammatory factors, and conducted comprehensive analyses of both pulmonary and gut microbiomes and metabolomics. Our findings indicate that exposure to PPT leads to significant pulmonary damage in these animals. The PPT group exhibited significantly elevated levels of total protein, albumin, alkaline phosphatase, and lactate dehydrogenase in bronchoalveolar lavage fluid, accompanied by marked upregulation of interleukin (IL)-18, tumor necrosis factor-alpha, IL-6, and IL-1β expression in lung tissue. Furthermore, 16S rRNA gene sequencing analysis revealed significant increases of *Akkermansia*, *Escherichia*-*Shigella*, and *Bacteroides* in both intestinal contents and lung tissue of PPT-treated animals, concomitant with notable elevations in short-chain fatty acids (SCFAs) such as isobutyric acid and isovaleric acid, and reductions in acetic acid, propionic acid, and butyric acid. The increased abundance of *Akkermansia* and *Escherichia*-*Shigella* may enhance pulmonary inflammatory factors through effects on intestinal barrier integrity and direct immune stimulation, while elevated *Bacteroides* may alter SCFA production, exacerbating pulmonary inflammation under PPT treatment, suggesting a potential role in the manifestation of PPT-induced pulmonary toxicity. This study offers new insights into the mechanisms of PPT-induced pulmonary toxicity, highlights the role of the gut-lung axis, and provides avenues for therapeutic intervention.

**IMPORTANCE:**

PPT, derived from the medicinal plant *Dysosma*, is known for its anti-cancer and anti-viral properties but limited by severe pulmonary toxicity. This study illuminates the gut-lung microbiota axis’s role in mediating this toxicity, revealing how specific microbial and metabolic alterations contribute to lung damage. By uncovering these mechanisms, our research opens avenues for interventions that could mitigate PPT’s side effects, potentially enhancing its safety and widening its therapeutic use.

## INTRODUCTION

Podophyllotoxin (PPT), a component of the aryltetralin lignans, is predominantly derived from plants such as *Dysosma* Woodson, *Diphylleia*, and *Podophyllum* spp. (1). Pharmacological studies have demonstrated that PPT exhibits multiple biological activities, including anti-viral and anti-inflammatory effects, and possesses DNA-damaging capabilities, alongside its anti-tumor actions (2). However, its significant toxicity and severe gastrointestinal side effects limit its clinical application. Elucidating its toxicological mechanisms provides a foundation for subsequent structural modification and the clinical application of new formulations (3).

The toxicity induced by PPT involves multiple organs, with the lungs being one of the crucial targets. As a significant active compound in *Podophyllum*, PPT has been linked to pulmonary damage. For instance, a clinical report noted acute respiratory failure in a patient following the ingestion of 50 mL of *Podophyllum-*medicated wine, highlighting its potential severity (4). Yet, the mechanisms underlying PPT’s pulmonary toxicity remain unclear, significantly hindering its clinical utility. Therefore, a systematic and comprehensive analytical approach is urgently needed to further explore its mechanisms of lung toxicity damage. In 2019, our team introduced the concept of the toxicological evidence chain (TEC) for the comprehensive evaluation of traditional Chinese medicine’s toxicity, which was detailed further in our subsequent publication (5). Accordingly, this project employs the TEC evaluation strategy, starting from a systematic toxicological assessment strategy of “harmful ingredient evidence,” “injury phenotype evidence (IPE),” “adverse outcome evidence (AOE),” “toxicological event evidence (TEE),” to clarify toxic evidence by identifying key elements at each link and creating cohesive connections between modules to establish a comprehensive toxicological evidence chain, exploring the biological mechanisms of PPT-induced pulmonary toxicity.

In recent years, extensive research into the correlations between gut microbiota and disease has revealed that various disorders are linked to disturbances within the gut microbiota, potentially triggering or exacerbating disease progression (6). The “gut-lung axis,” acting as a bidirectional communication channel between the respiratory and gastrointestinal systems, has garnered significant attention (7). Notably, gut microbiota and their metabolites have been proven to impact the functionality of the lung immune system through the gut-lung axis, contributing to the pathogenesis of several diseases, including chronic obstructive pulmonary disease (7), asthma (8), and cystic fibrosis (9). Our previous studies have demonstrated that PPT may alter the composition and function of the gut microbiota (10). However, it remains unclear whether these alterations in the gut microbiota, mediated by the gut-lung axis, participate in the development of PPT-induced pulmonary toxicity. The integrated application of high-throughput omics technologies provides guidance for a systematic and multifaceted toxicological evaluation.

In this study, employing targeted metabolomics, microbiome, and the TEC concept, we comprehensively elucidated the mechanisms of pulmonary toxicity induced by PPT. Using 16S rRNA gene sequencing, we detected structural and functional changes in microbial communities within rat intestinal contents and lung tissues. Additionally, gas chromatography-mass spectrometry (GC-MS) was utilized to assess alterations in short-chain fatty acids (SCFAs) in rat feces. Histological analysis with hematoxylin and eosin (H&E) staining and toxicity phenotype assessments were also conducted to evaluate the correlation between PPT-induced pulmonary toxicity and the gut-lung axis. Our findings demonstrate an association between PPT exposure and pulmonary toxicity, with alterations in the gut microbiota potentially correlating with the observed pulmonary effects. These results provide a basis for developing therapeutic strategies based on microbial or metabolite interventions.

## MATERIALS AND METHODS

### Animal and experimental design

#### Drugs and reagents

The compound podophyllotoxin (molecular formula: C_22_H_22_O_8_) was obtained from Shanghai Yuanye Biotechnology Company (China), under the item number B20477 and batch number A18GB145669. It was dissolved in dimethyl sulfoxide (D806647; Shanghai Macklin Biochemical Co., Ltd.) and sodium carboxymethyl cellulose (CP, 20120928; Shanghai Tiger Laboratory Equipment Co., Ltd.). For animal anesthesia, tribromoethanol (Avertin, 2401A; Nanjing Aibei Biotechnology Co., Ltd.) was used, while sodium chloride (AR, S24119; Shanghai Yuan Ye Biotechnology Co., Ltd.) and 4% paraformaldehyde (R20486, 500 mL; Shanghai Yuan Ye Biotechnology Co., Ltd.) were used for washing rat organs.

#### Animal handling

Forty-two SPF male Sprague-Dawley (SD) rats, weighing 210 ± 20 g, were housed in the animal laboratory of Henan University of Science and Technology First Affiliated Hospital. They were kept under controlled conditions with a 12 hour light-dark cycle, at a temperature of 23°C ± 2°C, and a humidity of 35% ± 5%, with 1 day of acclimation. Age- and weight-matched SD rats were randomly assigned to either the PPT solution group or the control (CON) group, with 22 rats in the PPT group and 20 in the control group. Rats in the PPT group were administered a daily dosage of 20 mg/kg of the PPT solution by intragastric (i.g.), while the control group rats were orally dosed with the vehicle solution for 4 days (Table S1).

#### Preparation of the PPT solution

The PPT solution was prepared at a concentration of 3 mg/mL according to the dosage. A precise amount of PPT was weighed and dissolved in a small volume of 2% dimethyl sulfoxide solution, then made up to volume with 0.5% CMC-Na solution, ultrasonicated for 1 hour, and stored at 4°C. The solution for the control group was identical except for the addition of PPT.

### Injury phenotype performance (IPE)

#### Observation of behavioral and physiological responses

After administration, we monitored both the behavioral and physiological changes in rats. This included observing symptoms such as diarrhea, bleeding, lethality, and changes in general behavior and appearance, such as color changes in the face, eyes, paws, and fur. Rat body weight was assessed and recorded daily. Additionally, gait scores were utilized to evaluate motor function and coordination by scoring the rats’ walking patterns on a scale from 0 (normal) to 4 (severe impairment), indicative of potential neurological or musculoskeletal distress. The lung organ index is calculated by dividing the weight of the lungs by the total body weight of the animal, providing a quantitative measure of potential inflammation, edema, or other pathological changes in the lungs. Changes in this index can indicate the impact of potential drug or other therapeutic interventions on lung health. The formula used is as follows: lung organ index = (lung weight / body weight) × 100, expressed as a percentage.

### Toxicological study on PPT-induced lung toxicity (AOE)

#### Determination of pro-inflammatory cytokines, oxidative stress, and biochemical markers

Levels of rat tumor necrosis factor-alpha (TNF-α) (MLBio, ml106471), interleukin (IL)-18 (MLBio, ml107045), IL-6 (MLBio, ml106838), and IL-1β (MLBio, ml106733) were determined using specific double-antibody sandwich ELISA kits from MLBio (Shanghai, China). To prepare lung tissue samples for the assay, samples were cut, weighed, homogenized in phosphate-buffered saline, and centrifuged, and the supernatant was collected for cytokine analysis. These prepared samples were then processed further as follows: purified monoclonal antibodies against each cytokine were coated on microtiter plate wells. After adding the tissue supernatants to these wells, a complex forms between the cytokine and horseradish peroxidase (HRP)-labeled detection antibodies. Following thorough washing to remove unbound components, 3,3′,5,5′-tetramethylbenzidine substrate was added; the color change induced by the HRP catalysis is quantitatively measured at 450 nm. This measurement directly corresponds to the cytokine levels in the samples. Furthermore, the malondialdehyde (MDA) content was determined at 532 nm using the thiobarbituric acid method, and reduced glutathione (GSH) levels were measured at 405 nm with the 5,5′-dithiobis (2-nitrobenzoic acid) method. Levels of total protein (TP), albumin (ALB), alkaline phosphatase (ALP), and lactate dehydrogenase (LDH) were quantified using a Thermo Scientific Indiko fully automatic biochemistry analyzer.

#### Histopathological study

The lungs of rats from each group were extracted and immersed in 4% paraformaldehyde for subsequent pathological section detection. Lung tissue was stained with H&E to observe lung tissue damage caused by PPT. H&E staining process involved dewaxing the paraffin sections with xylene, hydration, staining with hematoxylin for 10 minutes, differentiation, staining with eosin, dehydration, clearing in xylene, and sealing with neutral resin.

### Multiomics analysis of PPT-induced neurotoxicity (TEE)

#### 16S rRNA gene sequencing analysis

Fresh rat lung tissue and intestinal content samples were collected in sterile tubes, rapidly frozen in liquid nitrogen, and stored at −80°C until further processing. Total genomic DNA of the microbial communities was extracted from the samples, with the integrity of the extracted genomic DNA checked by 1% agarose gel electrophoresis. DNA concentration and purity were measured using a NanoDrop 2000 spectrophotometer (Thermo Scientific, USA). The extracted DNA served as a template for PCR amplification of the V3–V4 variable regions of the 16S rRNA gene, followed by sequencing library construction. High-throughput sequencing data were analyzed using the DADA2 plugin in QIIME2 software (2020 version, http://qiime2.org/), targeting the V3–V4 regions of the 16S rRNA gene to analyze bacterial taxa in lung tissue and intestinal contents. High-quality reads were identified as amplicon sequence variants (ASVs) with 100% sequence similarity and selected for bioinformatics analysis. After demultiplexing Illumina PE reads, the reads were quality controlled, filtered based on sequencing quality, and merged based on overlapping regions to obtain optimized data. Based on these, taxonomic classification, community diversity analysis, species difference analysis, and correlation analysis were conducted. Taxonomic assignment of ASVs was performed using the Naive Bayes consensus taxonomy classifier implemented in Qiime2 and the SILVA 16S rRNA database (v.138).

Alpha diversity, including abundance-based coverage estimator (ACE), Chao, and number of observed species (Sobs) indices, was compared using the Wilcoxon rank-sum test (11). Beta diversity analysis was conducted to assess the structural variation within microbial communities between the two groups. We measured the Bray-Curtis dissimilarity, a robust metric for quantifying the differences in community composition, and visualized the results using principal coordinate analysis (PCoA). Subsequently, we performed an analysis of similarities (Adonis) to confirm the significant differences in community structures. The microbial species composition at the genus level was evaluated for both groups. Bar charts and heatmaps identified differentially abundant taxa between groups. Differences in microbial community composition between the PPT and CON groups were identified using intergroup differential test methods. The linear discriminant analysis effect size (LEfSe) (http://huttenhower.sph.harvard.edu/LEfSe) was performed to identify the significantly abundant genus of bacteria among the different groups (linear discriminant analysis [LDA] score >3, *P* < 0.05). The correlation between differential microbial communities and environmental factors was further explored through heatmaps correlating differential microbes with inflammation markers MDA, GSH, and significantly different short-chain fatty acids. We focused our analysis on the most abundant bacterial species within each group, as these species are likely to exert significant physiological impacts. This approach also improves the reliability of our statistical analyses by minimizing the variability associated with less abundant species, which are often subject to higher detection and quantification errors.

#### Targeted metabolomics analysis of short-chain fatty acids

Metabolites from fecal samples were extracted and analyzed using GC-MS. Twenty milligrams of fecal sample was weighed into a 2 mL grinding tube, adding 800 µL of 0.5% phosphoric acid water (containing 10 µg/mL of internal standard 2-ethylbutyric acid), followed by freeze-grinding for 3 min (50 HZ), ultrasonication for 10 min, and centrifugation at 4°C and 13,000 × *g* for 15 min. Two hundred microliters of the supernatant was transferred to a 1.5 mL centrifuge tube, followed by the addition of 200 µL of butanol for extraction. The mixture was vortexed for 10 s, ultrasonicated at low temperature for 10 min, and centrifuged at 4°C and 13,000 × *g* for 5 min. The supernatant was then transferred to a sample vial for analysis.

The analytical instrument used was an Agilent Technologies 8890B-5977B GC/MSD system. Chromatographic conditions included an HP FFAP capillary column (30 m × 0.25 mm × 0.25 µm; Agilent J&W Scientific, Folsom, CA, USA), with high-purity helium gas (purity ≥99.999%) as the carrier gas at a flow rate of 1.0 mL/min. The injector temperature was 180°C, with a 1 µL injection volume, split injection with a split ratio of 10:1, and a solvent delay of 2.5 min. The temperature program started at 80°C, increased to 120°C at 20°C/min, then to 160°C at 5°C/min, and finally held at 220°C for 3 min. Mass spectrometry conditions included an electron impact ion source at 230°C, quadrupole at 150°C, transfer line at 230°C, and electron energy at 70 eV. The scanning mode was selected ion monitoring.

Automatic identification and integration of ion fragments were performed using the MassHunter Quantitative Analysis software (Agilent Technologies, v.10.0.707.0) with default parameters and manually checked. A linear regression standard curve was plotted with the analyte’s mass peak area as the *y*-axis and concentration as the *x*-axis. Sample concentrations were calculated by inserting the analyte’s mass peak area into the linear equation. Metabolites were annotated using the Kyoto Encyclopedia of Genes and Genomes (KEGG) (https://www.kegg.jp/) and The Human Metabolome Database (http://www.hmdb.ca/). Differences in metabolite concentrations between groups were analyzed using the *t*-test. Orthogonal partial least squares discriminant analysis was used for discriminant analysis of variables between different groups, calculating variable importance in projection (VIP) values. A VIP value of >1 and *P* < 0.05 were thresholds for selecting differential metabolites. Furthermore, KEGG pathway enrichment analysis was conducted on differential metabolites.

### Statistical analyses

Statistical analyses were conducted using SPSS (v.21; IBM, Armonk, NY, USA) and GraphPad Prism (v.8.0; GraphPad, CA, USA), with data presented as mean ± standard deviation. Data adhering to normal distribution and homogeneity of variance were analyzed using Student’s *t*-test, while non-parametric data were assessed with the Wilcoxon rank-sum test. To evaluate the diversity within microbial samples, alpha diversity indices, including Chao1, ACE, and Sobs, were calculated. Beta diversity was analyzed using the Bray-Curtis dissimilarity matrix and visualized through PCoA to illustrate differences in microbial community composition among samples. The LEfSe was employed to identify statistically significant microbial taxa between sample groups, pinpointing differentially abundant microbial markers and assessing their taxonomic contributions. Correlation network analysis was performed based on Spearman’s rank correlation, with significance determined for associations with an absolute correlation coefficient (|*r*|) greater than 0.5 and a *P* value less than 0.01, where a *P* value less than 0.05 was considered statistically significant.

The data presented in the study are deposited in the National Center for Biotechnology Information repository (accession numbers PRJNA1082651 and PRJNA1082519).

## RESULTS

### PPT-induced IPE

**Fig 1:**
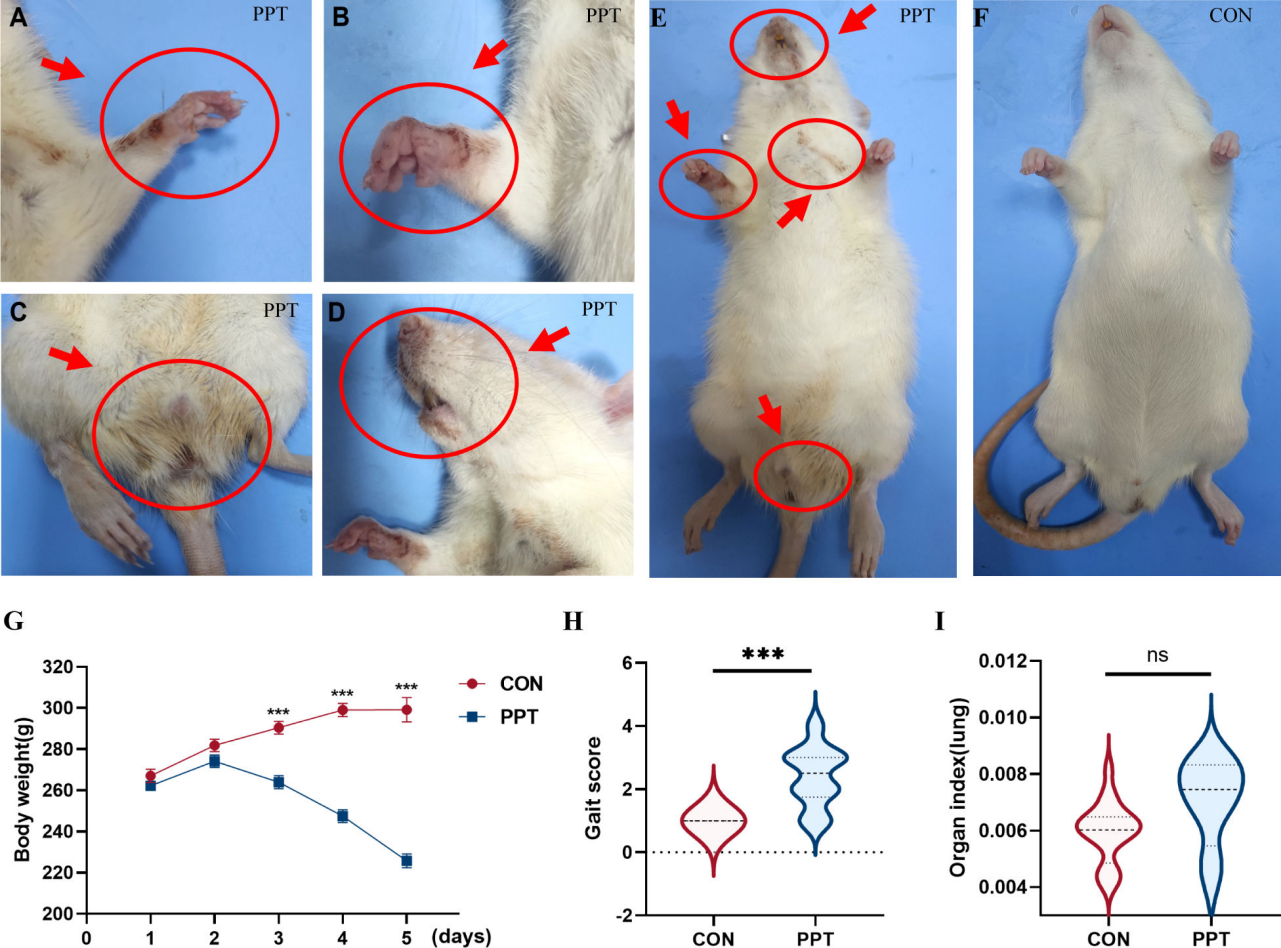
Physical and behavioral responses in SD rats following PPT administration. (**A–E**) Images depicting physical changes in PPT-treated rats: ecchymosis on forelimbs, bloodstains around the mouth and nose, fur darkening, and diarrhea. (**F**) Control (CON) group rat showing no signs of toxicity for comparison. (**G**) Gait score analysis assessing neurological impairments post-drug exposure, illustrating decreased mobility in PPT-treated rats. (**H**) Organ index comparison of lungs between PPT and CON groups, indicating no significant differences. (**I**) Longitudinal body weight tracking over 5 days post-exposure, showing significant weight loss in PPT-treated rats compared to controls. Data are presented as mean ± SEM. Significance is denoted by ***P < 0.001. ns, not significant.

To establish the physiological impact of PPT, we monitored specific injury phenotypes as immediate responses to drug exposure. Following drug administration, no significant changes were observed in the healthy control group. In contrast, on the third day post-administration, ecchymosis on forelimbs and bloodstains around the mouth and nose were observed in 12 rats (54.5%) from the PPT group. By day 4, all rats (100%) exhibited ecchymosis on their faces and paws, accompanied by significant changes in body weight and fur texture, indicating severe exposure effects. Two rats died 4 days after administration due to severe symptoms (Fig. 1A through E), while the control group rats showed no significant changes (Fig. 1F). Regarding body weight 4 days after administration, the control group maintained an average weight of 299.17 ± 5.87 g, while the PPT group showed a significant decrease to 225.77 ± 3.27 g (Fig. 1G). Gait scoring analysis revealed that all rats in the control group maintained a normal gait score of 1 (100%). In the PPT group, 4 rats (18.2%) maintained a normal gait; 3 rats (13.6%) showed slight abnormalities with a score of 2; 5 rats (22.7%) displayed moderate abnormalities with a score of 3; and 10 rats (45.5%) exhibited severe impairment with a score of 4, indicating significantly impaired motor abilities due to PPT exposure (Fig. 1H). The comparative analysis of lung organ indices indicated a trend toward an increase in the PPT group compared to the control group, yet the difference did not attain statistical significance (Fig. 1I). These observations confirm that PPT exposure significantly impairs physical health, as evidenced by the clear phenotypic manifestations of injury.

### Toxicological study on PPT-induced lung toxicity (AOE)

**Fig 2:**
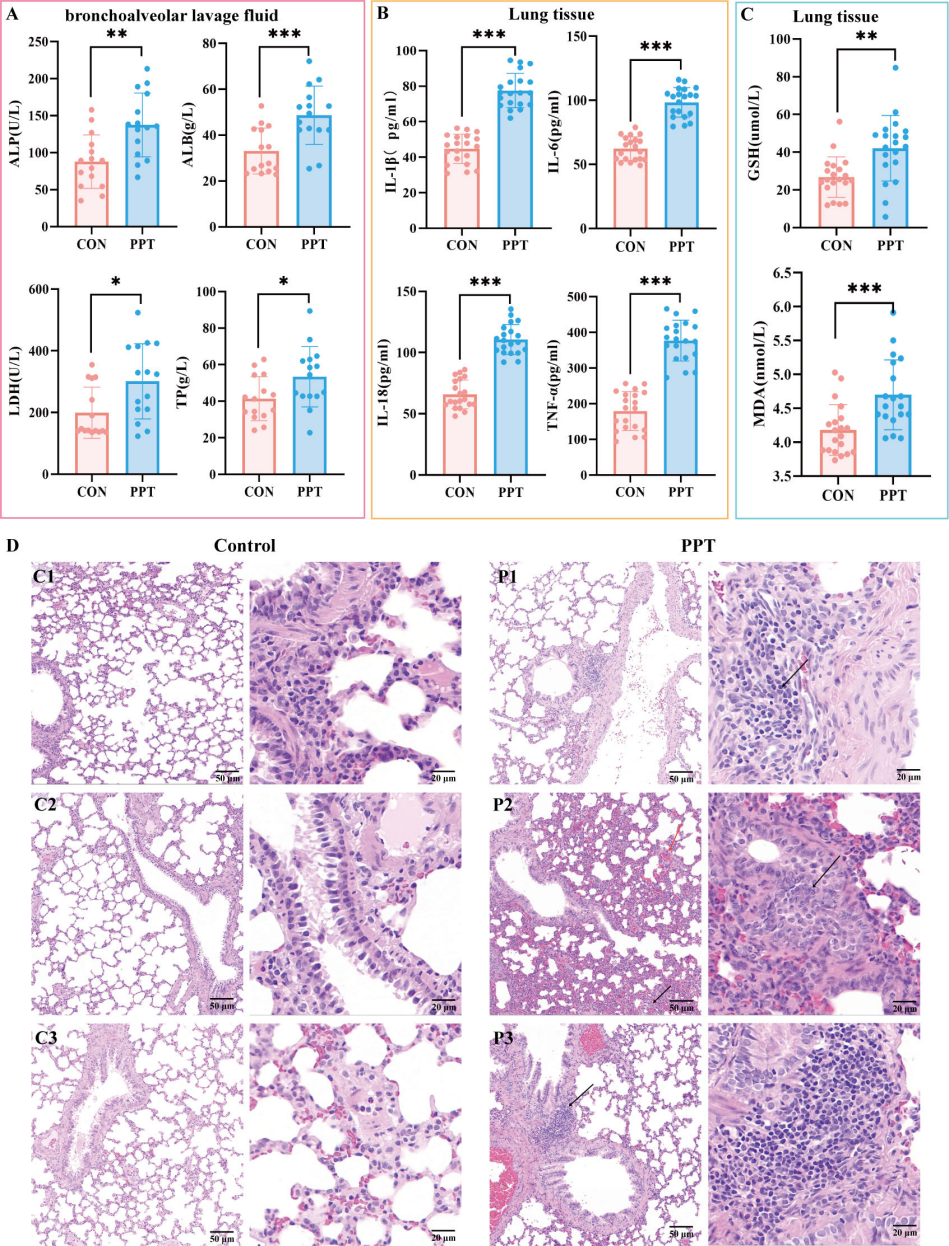
Comparison of biochemical, inflammatory, and oxidative stress markers in bronchoalveolar lavage fluid and lung tissue, along with histopathological changes in lung tissue in PPT-treated and control rats. (**A**) Biochemical markers in bronchoalveolar lavage fluid (ALP, ALB, LDH, and TP). (**B**) Inflammatory markers in lung tissue (IL -1β, IL-6, IL-18, and TNF-α). (**C**) Oxidative stress indicators in lung tissue (MDA and GSH). (**D**) Lung tissue was stained with H&E. Control group (C1–C3) showed normal architecture. PPT-treated group (P1–P3) had pathological changes, including lymphocyte infiltration (black arrows) and congestion (red arrows). Data are expressed as mean ± SEM. Significance levels are indicated as *P < 0.05, **P < 0.01, and ***P < 0.001. ns, not significant.

To elucidate the biochemical and pathological impacts of PPT on lung tissue, we conducted a comprehensive analysis of biomarkers and tissue pathology. In the bronchoalveolar lavage fluid of the PPT group, significant increases were observed in the levels of ALP, ALB, LDH, and TP (Fig. 2A). In lung tissue, pro-inflammatory cytokines, including IL-1β, IL-6, IL-18, and TNF-α, showed significant increases in the PPT group (Fig. 2B). In lung tissue, oxidative stress marker MDA significantly increased, along with a significant rise in reduced GSH (Fig. 2C). To further assess the pulmonary toxicity of PPT, lung tissue histopathology was examined using H&E staining. Compared to the healthy control group, which showed no significant pathological changes, the PPT group displayed pronounced pathological damage in lung tissue, including lymphocyte infiltration (black arrows), congestion (red arrows), capillary dilation and congestion in the alveolar walls, and a few red blood cells in the alveolar spaces (Fig. 2D). These pathological alterations, combined with biochemical and histological results, collectively suggest a strong inflammatory response and oxidative stress in lung tissues following PPT exposure.

### Pulmonary microflora disorder caused by PPT (TEE1)

**Fig 3:**
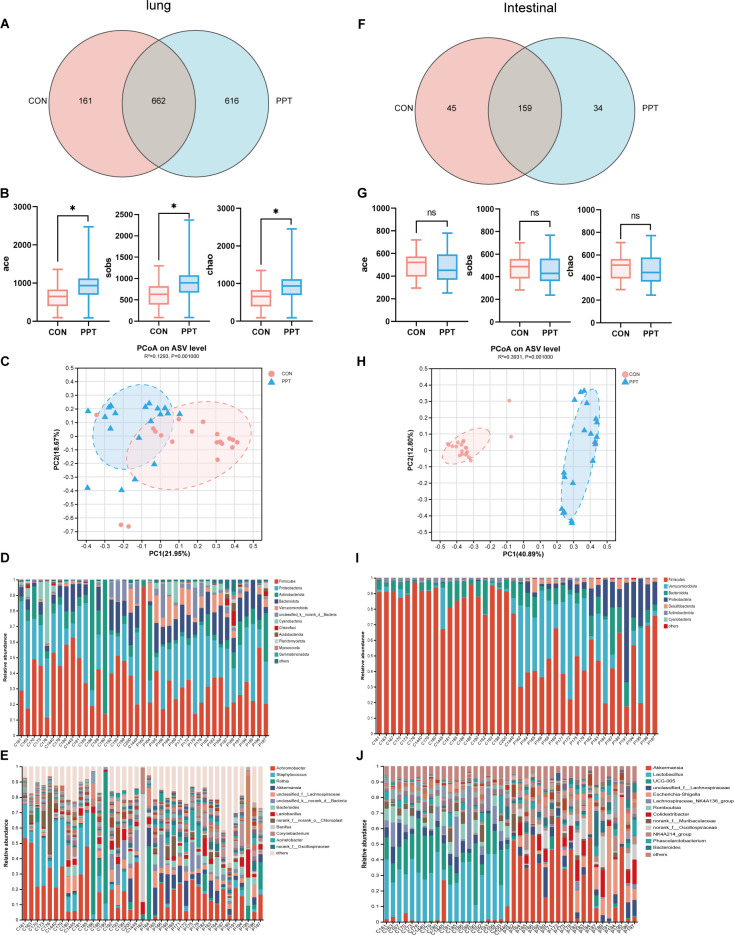
Microbial community analysis in rats subjected to PPT-induced changes in pulmonary and gut microbiota. (**A**) Venn diagram comparing the total and unique species in pulmonary microbiota between CON and PPT groups. (**B**) Alpha diversity indices (ACE, Chao, and Sobs) for pulmonary microbiota. (**C**) Principal coordinate analysis (PCoA) showing beta diversity in pulmonary microbiota on the ASV level. (**D and E**) Relative abundance and community composition of pulmonary microbiota across all samples at phylum (**D**) and genus (**E**) levels. (**F**) Venn diagram comparing the total and unique species in gut microbiota between CON and PPT groups. (**G**) Alpha diversity indices (ACE, Chao, Sobs) for gut microbiota. (**H**) PCoA illustrating beta diversity in gut microbiota on the ASV level. (**I and J**) Relative abundance and community composition of gut microbiota across all samples at phylum (**I**) and genus (**J**) levels. Data are presented as mean ± SEM. Significance levels are indicated as *P < 0.05. ns, not significant.

**Fig 4:**
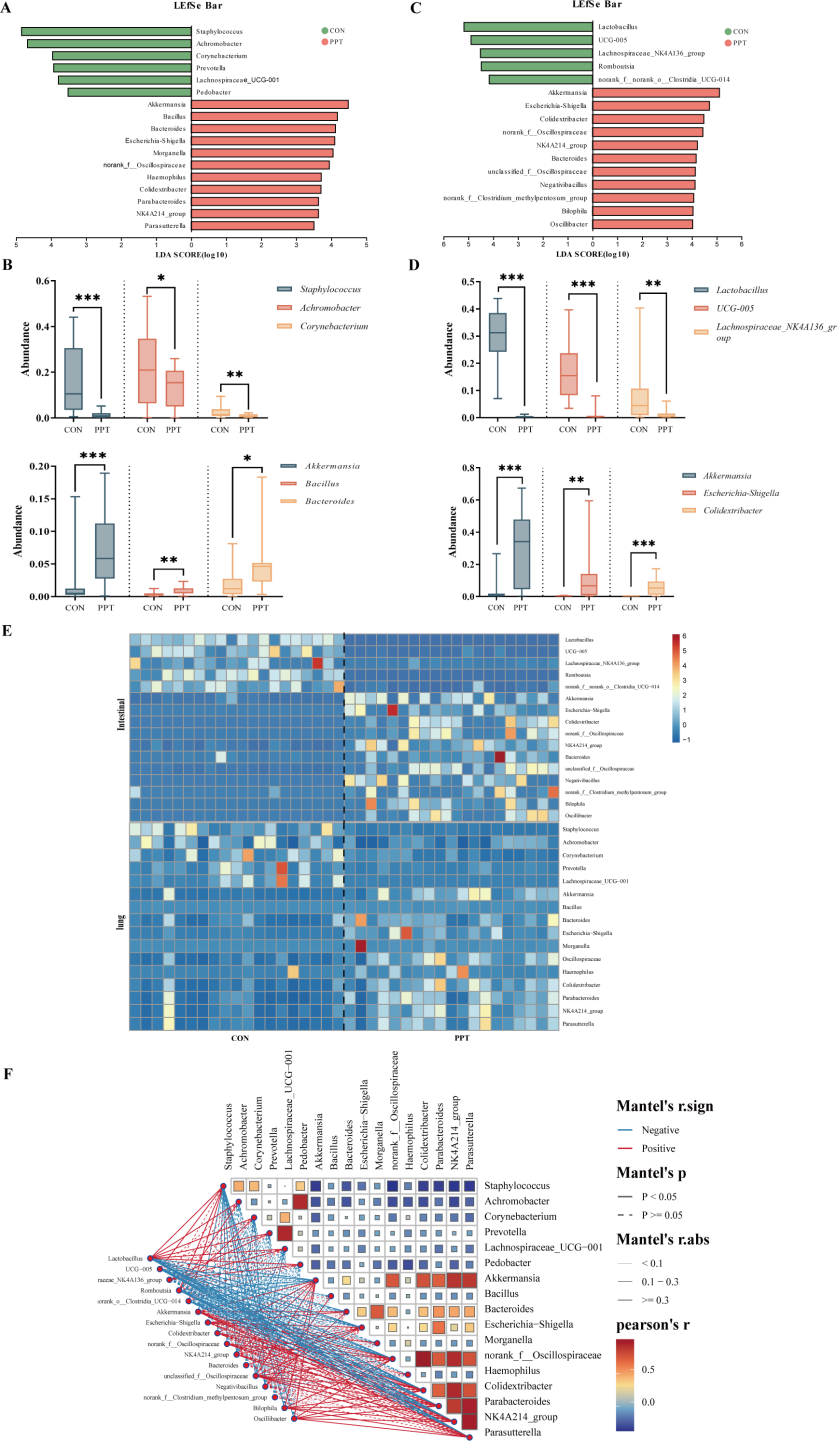
Comprehensive analysis of pulmonary and gut microbiota in rats subjected to PPT-induced pulmonary toxicity. (**A**) LEfSe analysis identifying significantly differentially abundant microbiota at the genus level in pulmonary samples. (**B**) Bar charts showing the relative abundance of significantly differentially expressed bacterial genera in the pulmonary samples. (**C**) LEfSe analysis highlighting significantly differentially abundant microbiota at the genus level in gut samples. (**D**) Bar charts depicting the relative abundance of significantly differentially expressed bacterial genera in the gut samples. (**E**) Heatmap illustrating the differential abundance of species in both pulmonary and intestinal samples. The color gradient extends from blue (low abundance) to red (high abundance). (**F**) Correlation heatmap depicting relationships within microbial communities of rat lungs and intestines after PPT treatment. Pairwise Pearson correlation coefficients between different bacterial genera are displayed, where colors range from blue (strong negative correlation) to red (strong positive correlation). The thickness of the lines represents the strength of the correlation, with solid lines indicating statistically significant correlations and dashed lines indicating no significant difference. Data are presented as mean ± SEM. Significance levels are indicated as *P < 0.05, **P < 0.01, and ***P < 0.001. ns, not significant.

16S rRNA gene sequencing of lung tissue samples from SD rats was conducted to characterize changes in pulmonary microbiota after PPT treatment. A Venn diagram showed that out of 1,439 identified genera, 616 were unique to the PPT group, 161 to the CON group, with 662 genera shared between groups, indicating that PPT may increase the richness of pulmonary microbiota (Fig. 3A). Alpha diversity indices, including ACE, Chao, and Sobs indices, were used to assess species richness, diversity, and coverage. The PPT group showed significantly higher ACE (*P* = 0.0236), Chao (*P* = 0.0238), and Sobs (*P* = 0.0287) compared to the CON group (Fig. 3B). PCoA demonstrated clear differences in microbial community structures between the CON and PPT groups, suggesting significant shifts due to PPT treatment (*R*²=0.1293, *P* = 0.001) (Fig. 3C). At the phylum level, *Firmicutes* dominated in both CON and PPT groups, with a noticeable decrease in *Actinobacteria* and a relative increase in *Proteobacteria* and *Bacteroidota* in the PPT group (Fig. 3D). At the genus level, *Achromobacter* and *Staphylococcus* dominated in the CON group, while *Akkermansia* and *Bacteroidota* were more abundant in the PPT group (Fig. 3E). We conducted LEfSe analysis on the pulmonary microbiota of the PPT and CON groups, revealing 17 significant differences in microbiota at the genus level (LDA >3.0, *P* < 0.05). *Akkermansia*, *Bacillus*, *Bacteroides*, and *Escherichia*-*Shigella* were found to be most abundant in the PPT group, while *Staphylococcus*, *Achromobacter*, *Corynebacterium*, and *Prevotella* predominated in the CON group (Fig. 4A). Analysis of the three most abundant bacterial species revealed higher levels of *Akkermansia*, *Bacillus*, and *Bacteroides* in the PPT group, in contrast to higher levels of *Staphylococcus*, *Achromobacter*, and *Corynebacterium* in the CON group (Fig. 4B). Moreover, a heatmap was constructed to show the abundance of all significantly different lung microbiota between the CON and PPT groups (Fig. 4E). These findings suggest an association between PPT exposure and a significant perturbation in the composition of the lung microbiota in SD rats.

### Intestinal flora disorder caused by PPT (TEE2)

Parallel to our pulmonary analysis, we assessed how PPT affects gut microbiota, given its critical role in overall health and immunity. 16S rRNA gene sequencing of intestinal content samples from SD rats was used to characterize changes in the gut microbiota after PPT treatment. A Venn diagram comparing the microbial composition and overlap between samples revealed a total of 238 identified genera, with 34 unique to the PPT group, 45 unique to the CON group, and 159 shared genera, indicating a potential reduction in gut microbiota richness by PPT (Fig. 3F). Despite using indices like ACE, Chao, and Sobs to assess diversity, no significant changes were detected (Fig. 3G). PCoAs showed distinct differences in gut microbiota structure and composition between CON and PPT groups (*R*²=0.3931, *P* = 0.001) (Fig. 3H). *Firmicutes* dominated in both groups at the phylum level, with a significant decrease in the PPT group compared to CON, whereas *Verrucomicrobiota*, *Bacteroidota*, and *Proteobacteria* were relatively more abundant in the PPT group (Fig. 3I). At the genus level, *Lactobacillus* dominated in the CON groups, with *Akkermansia* more abundant in PPT than in CON. (Fig. 3J). We conducted LEfSe analysis on the gut microbiota of the PPT and CON groups, revealing 16 significant differential microbiotas at the genus level (LDA >3.0, *P* < 0.05) with 5 enriched in the CON group and 11 in the PPT group. *Akkermansia*, *Escherichia*-*Shigella*, *Colidextricacter*, and *Bacteroides* were found to be most abundant in the PPT group, while *Lactobacillus*, *UCG-005*, *Lachnospiraceae-NK4A136_group*, and *Rmoboutsia* predominated in the CON group (Fig. 4C and D). A heatmap (Fig. 4E) illustrates PPT’s impact on microbial communities, notably increasing key microorganisms like *Akkermansia*, *Escherichia*-*Shigella*, and *Bacteroides* in both colon and lung tissues. The concurrent increase in key microorganisms like *Akkermansia*, *Escherichia*-*Shigella*, and *Bacteroides* suggests a synergistic role in the PPT-related changes affecting both the lung and intestinal microbiota. What is more, our analysis reveals significant correlations across these communities. There is evidence of significant correlations within both pulmonary and intestinal microbial communities, notably between *Akkermansia* and *Escherichia*-*Shigella* in both tissues. This correlation, along with a similar pattern observed between *Escherichia*-*Shigella* in the intestine and *Bacteroides* in the lung, indicates their potentially similar ecological roles within these microbiomes (Fig. 4F). The observed shifts in gut microbiota composition suggest a systemic effect of PPT, potentially influencing both local and distant physiological processes through the gut-lung axis.

### PPT-induced changes in metabolic pathways (TEE3)

**Fig 5:**
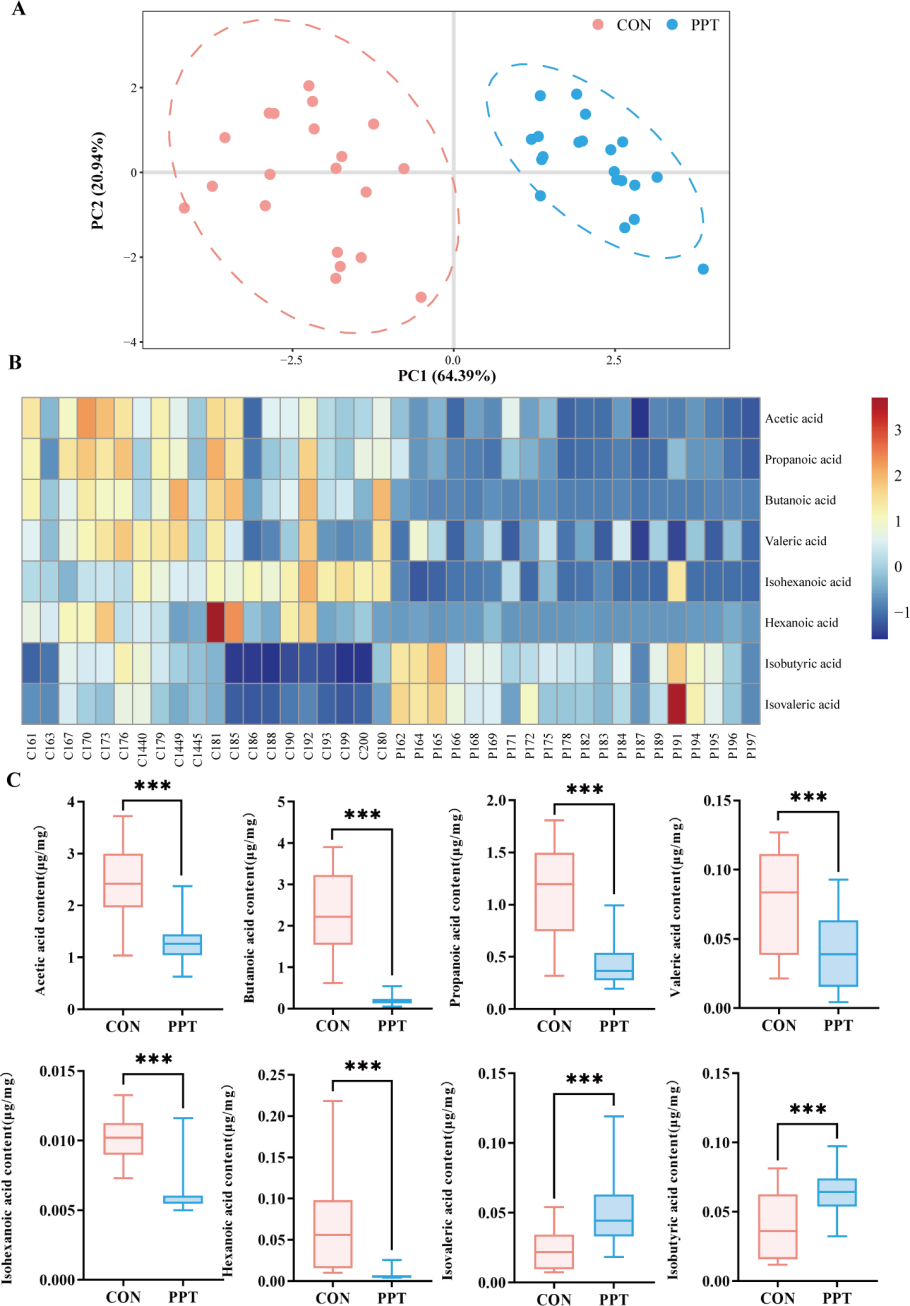
Targeted quantitative analysis of short-chain fatty acids (SCFAs) in rats subjected to PPT-induced pulmonary toxicity. (**A**) PLS-DA scatter plot with each point representing a rat sample, color-coded by group: blue for control (CON) and red for PPT-treated, indicating the variance explained by the first two principal components. (**B**) Heatmap depicting the concentration levels of various SCFAs across samples, with color intensity varying from blue for low concentrations to red for high concentrations. (**C**) Box plots comparing the concentrations of eight specific SCFAs between the CON and PPT groups, with significance levels marked by asterisks (***P < 0.001).

To further understand the metabolic consequences of microbiota disruption, we quantified key metabolites, focusing on SCFAs that play essential roles in immune regulation and barrier function (12, 13). The concentrations of target substances in the samples were determined using GC-MS detection and standard curve calculations. Partial least squares discriminant analysis (PLS-DA) demonstrated clear separation between the PPT group and the control group, indicating significant differences in cecal feces SCFAs between the two groups (Fig. 5A). Correlation heatmaps showed significant changes in eight SCFAs, with isobutyric acid and isovaleric acid being higher in the PPT group, and acetic acid, propanoic acid, butanoic acid, valeric acid, isohexanoic acid, and hexanoic acid showing significant decreases (Fig. 5B and C). KEGG pathway analysis based on differential metabolites identified 14 metabolic pathways, with protein digestion and absorption, carbohydrate digestion and absorption, glycosaminoglycan biosynthesis (heparan sulfate/heparin), and alcoholic liver disease showing high impact values (Fig. S1). The alterations in SCFA profiles underscore the metabolic disruption caused by PPT, linking changes in microbiota to functional metabolic outcomes.

### Co-occurrence analysis among gut microbiota, pulmonary microbiota, metabolites, biochemical markers, and inflammatory cytokines (TEE4)

**Fig 6:**
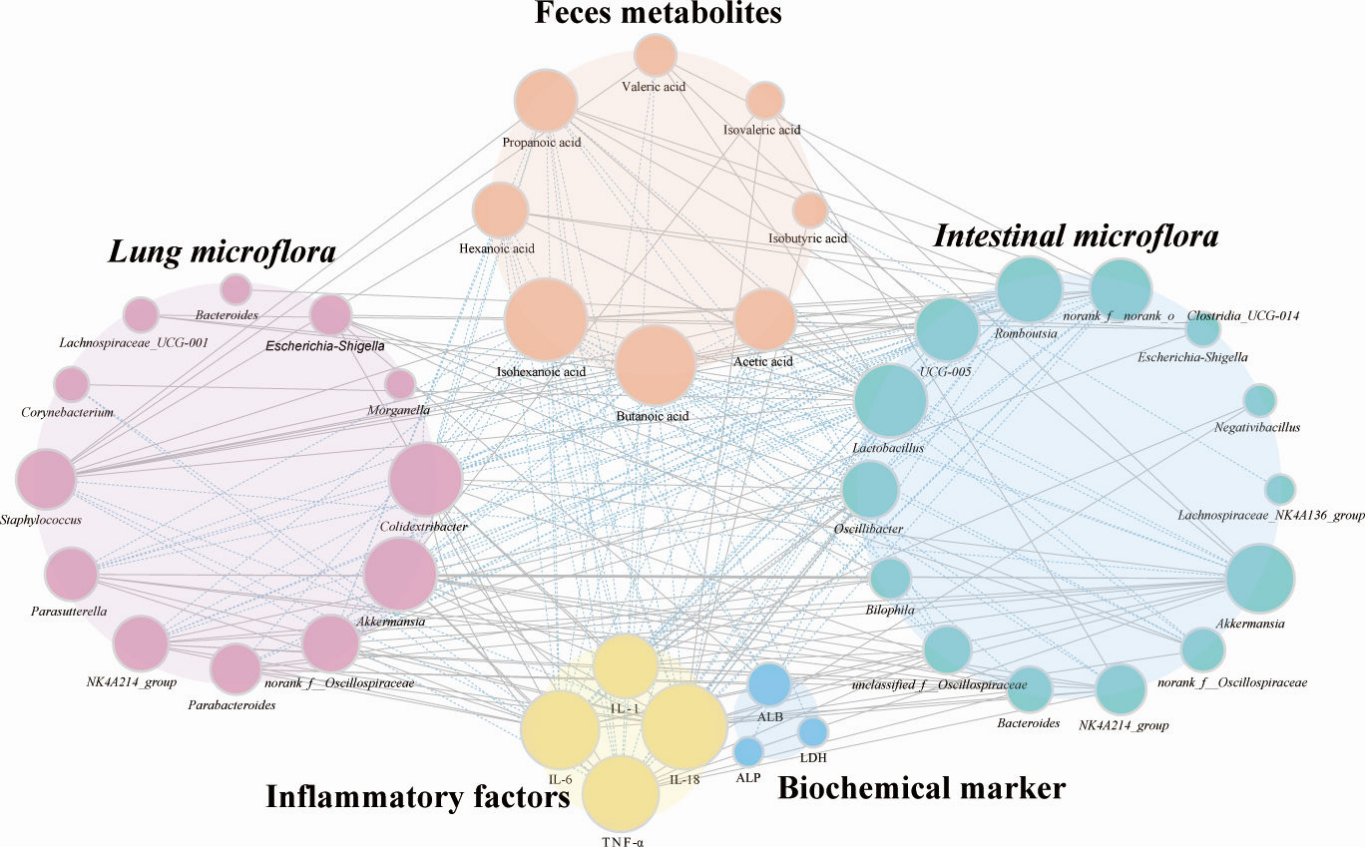
Co-occurrence network analysis. The diagram illustrates the relationships among microbial genera from lung and gut microbiomes, metabolites, biochemical markers, and pro-inflammatory cytokines. Node size represents the importance or abundance of each variable. Blue lines represent negative Spearman correlations, while gray lines indicate positive correlations. The thickness of the lines reflects the strength of each correlation.

To explore the complex interrelationships among gut microbiota, pulmonary microbiota, SCFA metabolites, and inflammatory cytokines, Spearman correlation analysis was performed on the differential substances. The co-occurrence network revealed five main interconnected clusters, including the top 17 abundant pulmonary differential microbial groups, top 16 gut differential microbial groups, 8 significantly different SCFA metabolites, 4 biochemical markers, and 4 inflammatory factors. Key elements in these clusters included SCFA metabolites such as isohexanoic acid, butanoic acid, acetic acid, and propanoic acid; gut microbiota like *Lactobacillus*, *Akkermansia*, *Romboutsia*, and *UCG-005*; pulmonary microbiota such as *Akkermansia*, *Colidextribacter*, and *Staphylococcus*; and the biochemical marker ALB, along with inflammatory factors IL-6, TNF-α, and IL-1β, all playing significant roles in the network. Notably, *Akkermansia*, present in both lung and intestines, exhibits a positive correlation with the inflammatory markers IL-6, TNF-α, IL-1β and is also positively correlated with isovaleric acid and isobutyric acid. Intestinal microbes *Bacteroides* spp. are positively correlated with IL-18, TNF-α, and butanoic acid. Additionally, IL-18 is positively correlated with isovaleric acid and isobutyric acid (Fig. 6). These results emphasize the importance of the gut-lung axis in the pulmonary toxicity of PPT, demonstrating the complex interactions and potential functional roles of these microbiota and metabolites in physiological and pathological processes.

## DISCUSSION

PPT is a lignan derivative widely found in plants of the family Berberidaceae, including species of *Dysosma*, *Diphylleia*, and *Sinopodophyllum*, known as “podophyllotoxin source plants” (14). PPT has been shown to possess various clinical effects, such as anti-viral (15), anti-tumor (16), anti-cancer (17), insecticidal (18), apoptosis-inducing (19), and immunotherapeutic activities (20). However, its narrow therapeutic window and organ toxicity, including effects on the lungs and the gastrointestinal tract, limit its clinical application. Several cases of poisoning and even death after ingestion of PPT have been reported clinically, with a low chance of successful resuscitation, and symptoms vary with the degree of poisoning. Thus, elucidating the underlying mechanisms of PPT-induced pulmonary toxicity is crucial for the safe use of PPT, potential therapeutic applications, and repositioning of PPT-based drugs. It is important to note that this study is observational, and the current results only reveal associations between PPT exposure, changes in the gut microbiota, and lung toxicity. The roles of the microbiome and the gut-lung axis in the process of PPT-induced lung toxicity have not been fully validated. The subsequent discussion will focus on the existing observational results and make reasonable inferences about potential mechanisms.

Our findings indicate that, compared to the control group, rats in the PPT group exhibited varying degrees of bleeding and ecchymosis on their faces and paws, and their fur turned brownish. The systemic effects, including changes in organ indices, body weight, and gait scores, align with our previous research that documented system damage from PPT exposure (10, 21). These findings validate the broader systemic impact of PPT. However, our primary focus in this study is on pulmonary toxicity. Substantial evidence of lung damage is demonstrated by H&E staining, which revealed significant pathological changes such as lymphocyte infiltration and congestion in the lungs, underscoring the critical nature of pulmonary responses to PPT exposure. Biochemical markers, including ALB, ALP, TP, and LDH, were significantly increased, indicating tissue damage, particularly to the lungs, where increased LDH activity suggests severe injury (22). The increase in TP content indicates damage to the pulmonary vascular wall, increased permeability, and protein entry into the alveolar cavity. Pro-inflammatory markers IL-1β, IL-6, TNF-α, and IL-18 were significantly increased. TNF-α, an important cytokine in early inflammatory responses, can directly damage pulmonary vascular endothelial cells and alveolar type II epithelial cells, promoting inflammation spread in lung tissue (23). The significant increase in TNF-α suggests a certain degree of inflammatory damage to lung tissue (24). The simultaneous overexpression of TNF-α and IL-6 in PPT-exposed rats indicates immune system disorder and severe damage in lung tissue (25). The significant rise in the oxidative stress marker MDA indicates lung damage due to oxidative stress from PPT, with an increase in GSH suggesting a systemic compensatory response to oxidative damage (26). Certain pathological conditions may promote both the generation of MDA and the upregulation of GSH, reflecting the dynamic balance between cell damage and protection.

In recent years, the study of the lung-gut axis has gradually increased, revealing the complex relationship between gut microbiota and lung health. The concept of the lung-gut axis emphasizes the bidirectional communication between the gut and lungs, which may have important effects on the pathophysiological processes of many diseases. Dysbiosis of certain gut microorganisms can lead to abnormal immune responses, thereby exacerbating symptoms of lung disease (27). Additionally, metabolites from the gut microbiota may also affect inflammatory responses and immune regulation in the lungs through the bloodstream (28). To investigate whether the pulmonary toxicity of PPT is related to the lung-gut axis, we carried out multiomics analysis.

After PPT exposure, similar trends in changes were observed in both lung and gut microbiota, with a synchronous increase in the relative abundance of *Bacteroidota* and *Proteobacteria*. However, based on the current evidence of this study, it cannot be definitively concluded that PPT is the direct cause of these changes. PPT significantly increased the abundance of lung microbiota, with *Firmicute*s dominating in both CON and PPT groups, a decrease in *Actinobacteri*a, and an increase in *Proteobacteria* and *Bacteroidota* in the PPT group. *Proteobacteria*-associated dysbiosis correlates with inflammatory profiles, low alveolar macrophages, and high neutrophils in bronchoalveolar lavage fluid. Dysbiosis driven by *Bacteroidota* is often associated with a pro-remodeling cell activation profile, high alveolar macrophages, and low neutrophils percentage (29). *Firmicutes*, a group containing many beneficial bacteria producing SCFAs such as acetate and lactate, beneficially affects the intestinal environment, modulates the symbiotic microbiota ratio, alleviates inflammation, and regulates immune balance, enhancing the interaction between gut microbiota and host. PPT exposure significantly reduced the abundance of *Firmicutes. Proteobacteria*, containing various pathogenic species like *Escherichia coli*, are considered a microbial marker of gut microbiota dysbiosis (30). We evaluated the composition of microbial species in both groups at the genus level. Notably, there was a concurrent increase in the relative abundance of *Akkermansia*, *Escherichia*-*Shigella*, and *Bacteroides* in both the pulmonary and gut microbiota. *Akkermansia*, known for its mucin-degrading capabilities, can affect intestinal barrier function and potentially enhance intestinal permeability (31). Increased intestinal permeability may allow more microbes and metabolites such as SCFAs to enter the bloodstream, impacting distant organs like the lungs. Furthermore, *Escherichia*-*Shigella*, capable of producing endotoxins, can provoke the host’s immune response and amplify the release of inflammatory signaling molecules, including IL-1β and TNF-α. These factors may induce inflammatory responses within the lungs (32). *Bacteroides* sp. plays a key role in immune modulation and intestinal health by producing a variety of SCFAs, including propionate and acetate (33). The enrichment of *Akkermansia*, *Escherichia*-*Shigella*, and *Bacteroides* post-PPT underscores the lung-gut axis’s role in modulating immune functions and addressing gut microbiota imbalance.

Recent studies have found that dysbiosis of gut microbiota is associated with various acute and chronic inflammatory lung diseases, and gut microbiota disturbances can induce or exacerbate lung inflammation (34). SCFAs, as the main metabolites of gut microbiota, play crucial roles in maintaining the intestinal epithelial cell barrier, regulating host immunity, and maintaining the dynamic balance of oxidative-anti-oxidative reactions (35). Literature reports that supplementing the diet of mice infected with influenza virus with high fiber to enhance gut SCFAs effectively improved mouse survival rates and mitigated airway inflammation, suggesting that improving gut microbiota structure and abundance benefits lung immunity and pneumonia. SCFAs can enter lung tissue via the circulatory system, activating receptors on immune and epithelial cells, regulating chemotaxis of neutrophils and T-cell proliferation, and inhibiting the nuclear factor kappa-light-chain-enhancer of activated B cell signaling pathway to reduce inflammation (36). Our study measured SCFAs in rat cecal feces, finding significant increases in potentially harmful isobutyric and isovaleric acids, which might modulate lung immune functions by activating G protein-coupled receptors like GPR41 and GPR43. This activation could promote the proliferation and activation of macrophages and T cells, thereby increasing the expression of IL-6 and TNF-α (37). The results suggest an association between PPT exposure and a significant decrease in the concentrations of beneficial SCFAs such as acetic acid, propanoic acid, butanoic acid, valeric acid, isohexanoic acid, and hexanoic acid. A decrease in these SCFAs might be associated with a diminished protective effect, potentially related to a more intense pulmonary response to PPT (37). Research confirms that SCFAs from gut microbiota alleviate lung injury in severe acute pancreatitis in mice (38), and propionate and acetate significantly protect against nano-zinc oxide-induced acute lung injury and ischemia-reperfusion lung injury (39, 40). Our findings indicate that beneficial SCFA metabolites significantly decreased after PPT exposure, suggesting that PPT may mediate pulmonary inflammatory damage and toxicity through its impact on SCFA metabolism.

Microorganisms regulate physiological functions through their metabolites, a primary pathway by which the microbiome exerts its effects. This study analyzed the correlation between fecal metabolites and lung and gut microbiota post-PPT exposure. The abundance of *Akkermansia*, *Escherichia*-*Shigella*, and *Bacteroides* in the lung and gut microbiota in the PPT exposure group was significantly higher than that in the control group, and there was a strong positive correlation between *Akkermansia* in the lungs and *Akkermansia* and *Bacteroides* in the intestine. *Akkermansia*’s increase correlates with elevated inflammatory markers like IL-6, TNF-α, IL-1β, and IL-18 in both sites, suggesting it may boost inflammatory factor production by compromising the intestinal barrier and directly stimulating immune responses, thus promoting lung inflammation (41). In addition, *Akkermansia* in the intestine is also associated with isovaleric acid and isobutyric acid. There is a positive correlation between *Bacteroides* and butanoic acid, and the increase in *Bacteroides* may affect the immune balance of the lungs by regulating the production of SCFA. *Bacteroides* can produce anti-inflammatory SCFA, but under PPT treatment, the balance of its production may be disrupted, resulting in a decrease in anti-inflammatory SCFA and an increase in pro-inflammatory SCFA, thereby exacerbating lung inflammation (32). When the intestinal barrier is compromised by factors like diseases, drugs like PPT, or other inflammatory conditions, microbial translocation may occur, allowing microorganisms or their components (e.g., endotoxins and metabolites) to enter the lungs via the bloodstream (42). This process might enable originally intestinal microorganisms to survive and proliferate in the lungs, becoming predominant species in both lung and gut contents. In summary, this study found that after PPT treatment, there was an increase in the abundance of *Akkermansia*, *Escherichia*-*Shigella*, and *Bacteroides* in both gut and lung tissues, accompanied by an increase in harmful SCFAs, such as isobutyrate and isovalerate, and a decrease in beneficial SCFAs. Based on these results, it is speculated that these microbes may participate in the development of lung toxicity through mechanisms involving gut barrier function, immune stimulation, and SCFA metabolism. It is important to note that the current study primarily reveals associations between PPT exposure, microbiota dysbiosis, and pulmonary toxicity. While these findings highlight potential pathways via the gut-lung axis, causal relationships and mechanistic details require further experimental validation, such as fecal microbiota transplantation or targeted metabolite supplementation in future studies.

### Conclusion

This study, based on the gut-lung axis concept, comprehensively explores the potential toxicological mechanisms of PPT-induced pulmonary toxicity. Experimental results show that the increase in TP, ALB, ALP, and LDH levels in the bronchoalveolar lavage fluid of rats, as well as the increase in MDA and GSH levels in lung tissue, indicates that PPT may cause pulmonary pathological changes by damaging cellular integrity and inducing oxidative stress responses in the lungs. Moreover, PPT not only disrupts the homeostasis of the gut microbiota but also affects the abnormal content of its metabolites, leading to lung homeostasis disorder and making the host susceptible to or exacerbating lung injury. Particularly, the simultaneous increase in *Akkermansia*, *Escherichia*-*Shigella*, and *Bacteroides* in the colonic contents and lung tissue may further trigger pulmonary inflammatory responses and damage by affecting the metabolism of SCFAs (such as increased isobutyric acid and isovaleric acid) and the production of other inflammatory mediators (such as IL-18, TNF-α, IL-6, and IL-1β). These results suggest that PPT exposure was correlated with inflammation and pulmonary toxicity, potentially linked to the enrichment of specific bacterial taxa. In summary, this study provides a new theoretical perspective for early intervention in PPT-induced pulmonary toxicity and identifies potential bacterial or metabolite targets, deepening the understanding of the toxicological mechanisms related to PPT and offering theoretical guidance for further exploration of strategies to mitigate the toxicity and enhance the efficacy of PPT.

## Data Availability

The data presented in the study are deposited in the National Center for Biotechnology Information repository (accession numbers PRJNA1082651 and PRJNA1082519).

## SUPPLEMENTAL MATERIAL

Supplemental figure and table spectrum.01653-24-s0001.docx Figure S1 and Table S1.

ASM does not own the copyrights to Supplemental Material that may be linked to, or accessed through, an article. The authors have granted ASM a non-exclusive, world-wide license to publish the Supplemental Material files. Please contact the corresponding author directly for reuse.
