# Supplementary material for: Characterizing the impact of podophyllotoxin on pulmonary toxicity and gut-lung microbiota interactions in SD rats based on TEC concept
Source: Microbiol Spectr. 2025 Apr 25;13(6):e01653-24. doi: 10.1128/spectrum.01653-24 (PMC12131824; doi:10.1128/spectrum.01653-24)
Supplement: Supplemental figure and table — Figure S1 and Table S1. [file spectrum.01653-24-s0001.docx]

**Supplementary Figure 1**. KEGG enrichment analysis of eight short-chain fatty acids with significant differences between PPT group and CON group.


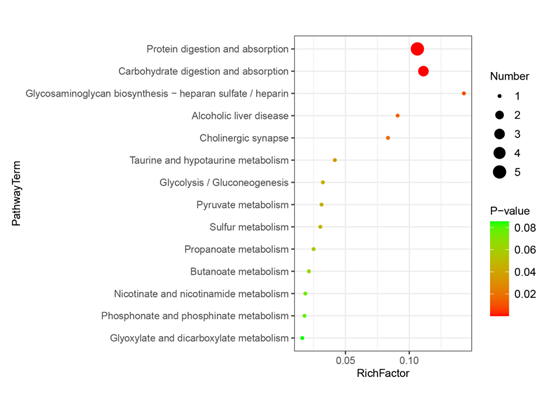


**Supplementary Table1.** Administration scheme of PPT on pulmonary toxicity in rats.

| **Group** | **Number** | **Drug** | **Dose** | **Administration method** | **Exposure period** |
| --- | --- | --- | --- | --- | --- |
| **CON** | 20 | 2%DMSO+ 0.5%CMC-Na | 2 mL/d | i.g. | 4 d |
| **PPT** | 22 | 2%DMSO +0.5% CMC-Na+ PPT | 20 mg/kg/d |  | 4 d |

i.g., gavage.
